# Structural brain network analysis in occipital lobe epilepsy

**DOI:** 10.1186/s12883-023-03326-z

**Published:** 2023-07-15

**Authors:** Dong Ah Lee, Ho-Joon Lee, Kang Min Park

**Affiliations:** 1 Department of Neurology, Haeundae Paik Hospital, Inje University College of Medicine, Busan, Korea; 2 Department of Radiology, Haeundae Paik Hospital, Inje University College of Medicine, Busan, Korea

**Keywords:** Diffusion tensor imaging, Epilepsy, Occipital lobe

## Abstract

**Background:**

This study aimed to analyze the structural brain network in patients with occipital lobe epilepsy (OLE) and investigate the differences in structural brain networks between patients with OLE and healthy controls.

**Methods:**

Patients with OLE and healthy controls with normal brain MRI findings were enrolled. They underwent diffusion tensor imaging using a 3.0T MRI scanner, and we computed the network measures of global and local structural networks in patients with OLE and healthy controls using the DSI studio program. We compared network measures between the groups.

**Results:**

We enrolled 23 patients with OLE and 42 healthy controls. There were significant differences in the global structural network between patients with OLE and healthy controls. The assortativity coefficient (-0.0864 vs. -0.0814, *p* = 0.0214), mean clustering coefficient (0.0061 vs. 0.0064, *p* = 0.0203), global efficiency (0.0315 vs. 0.0353, *p* = 0.0086), and small-worldness index (0.0001 vs. 0.0001, *p* = 0.0175) were lower, whereas the characteristic path length (59.2724 vs. 53.4684, *p* = 0.0120) was higher in patients with OLE than those in the healthy controls. There were several nodes beyond the occipital lobe that showed significant differences in the local structural network between the groups. In addition, the assortativity coefficient was negatively correlated with the duration of epilepsy (r=-0.676, *p* = 0.001).

**Supplementary Information:**

The online version contains supplementary material available at 10.1186/s12883-023-03326-z.

## Significance

We first evaluated alterations in the structural brain network of patients with OLE. There were significant differences in the global structural network between patients with OLE and healthy controls. We also found several regions of nodes that showed significantly altered local structural networks in patients with OLE. This study could suggest that epilepsy is a network disease, and this also may apply to OLE.

**Supplementary Information:**

The online version contains supplementary material available at 10.1186/s12883-023-03326-z.

## Introduction

Occipital lobe epilepsy (OLE) is uncommon, constituting about 5% of cases of focal epilepsy [1, 2]. The ictal clinical manifestations of OLE include visual subjective symptoms such as visual hallucinations, blindness, visual illusions, palinopsia, and sensory hallucinations of ocular movements. Subjective ocular symptoms comprise ocular pain, whereas objective oculomotor symptoms include tonic deviation of the eyes, oculo-clonic movements or nystagmus, and repetitive eyelid closure or eyelid fluttering [1, 3].

Network analysis has been introduced recently into the neuroscience field and has helped to elucidate the etiology of various neurological diseases, including epilepsy [4, 5]. Furthermore, network analysis not only reveals the pathogenesis of epilepsy but is also advancing clinical practice by aiding in localization or lateralization of epileptogenic lesions, [6, 7] prediction of anti-seizure medication (ASM) response, [8–10] and expectation of outcome after epilepsy surgery [11]. In addition, with the assistance of machine learning or deep learning, unraveling the brain network and connectivity will play an important role in the management of patients with epilepsy in the future [12, 13]. However, the previous studies analyzing brain networks in patients with epilepsy have enrolled patients with focal epilepsy originating from all lobes or have focused on temporal lobe epilepsy, a common focal epilepsy [10, 11, 14–16]. Although some studies have also investigated the brain network in patients with frontal lobe epilepsy, [17, 18] there are no studies on brain network analysis focusing on patients with OLE.

The human brain is one of the world’s most complex networks, and research on its static and dynamic properties has exploded in recent years. Advancements in graph theory and network neuroscience provide the opportunity to comprehend the complexities of this phenomenon and its modeling [19, 20]. Graph theoretical methods have established a mathematical framework for modeling pairwise communications between network nodes. Graph theory is typically applied to either structural or functional networks in human neuroscience [19, 20]. Structural networks, which measure white matter integrity and functional networks, and measures of coupling strength, appear to exhibit positive correlations in the healthy adult brain, in that regions of the brain that are highly structurally interconnected tend to exhibit strong patterns of functional connectivity [20]. Recent advances in magnetic resonance imaging (MRI) and image processing provide numerous non-invasive methods for quantifying structural networks using short-range local measures and/or long-range fiber tract tracing techniques, known as diffusion tractography based on diffusion tensor imaging (DTI) [20].

In this study, we analyzed the structural brain network in patients with OLE using DTI and graph theory and investigated the differences in structural brain networks between patients with OLE and healthy controls. We hypothesized that there would be significant alterations in the structural brain network in patients with OLE compared to healthy controls. This is the first study to focus on the structural brain networks in OLE.

## Materials and methods

### Participants

The Institutional Review Board of our university authorized this research. This retrospective examination was performed at a tertiary hospital. The following inclusion criteria were applied to enroll newly diagnosed patients with OLE: [1, 3, 21] (1) seizure semiology typical of OLE, (2) ictal EEG originating in the occipital lobe, and (3) underwent DTI at the time of epilepsy diagnosis. We collected data on age, sex, age at seizure onset, and duration of epilepsy for each patient (time from seizure onset to MRI). We excluded participants who had any other abnormalities visible on brain MRI or who had any medical disease, such as diabetes mellitus or hypertension, or neurological diseases, such as ischemic stroke, encephalitis, or any injury, apart from epilepsy.

We also enlisted age- and sex-matched healthy controls with no history of medical or neurological disorders from a database of controls. They had already been recruited for our previous study [22]. All healthy controls had normal MRI scans of the brain.

### MRI acquisition

Patients with OLE and healthy controls underwent DTI and three-dimensional T1-weighted imaging using a 3.0T MRI scanner equipped with a 32-channel head coil (AchievaTx, Phillips Healthcare, Best, The Netherlands). In a recent study, we previously described the DTI data processing and analysis [4]. The specific DTI parameters were as follows: 32 different diffusion directions, b-values of 0 and 1,000 s/mm^2^ (b0 images were acquired once), TR/TE = 8620/85 ms, FA = 90°, slice thickness = 2.25 mm, acquisition matrix = 120 × 120, field of view = 240 × 240 mm^2^, parallel imaging factor (SENSE) of 2. The phase direction was set to the anterior-posterior direction, and the fat was shifted posteriorly. The T1-weighted images were acquired using a turbo-field echo sequence with the following parameters: TI = 1300 ms, TR/TE = 8.6/3.96 ms, flip angle = 8°, and voxel size of 1 mm^3^. The MR sequences also included three-dimensional fluid-attenuated inversion recovery and coronal T2-weighted imaging, which were used to evaluate the structural lesions in the participants with visual inspections.

### Structural network analysis

**Fig. 1:**
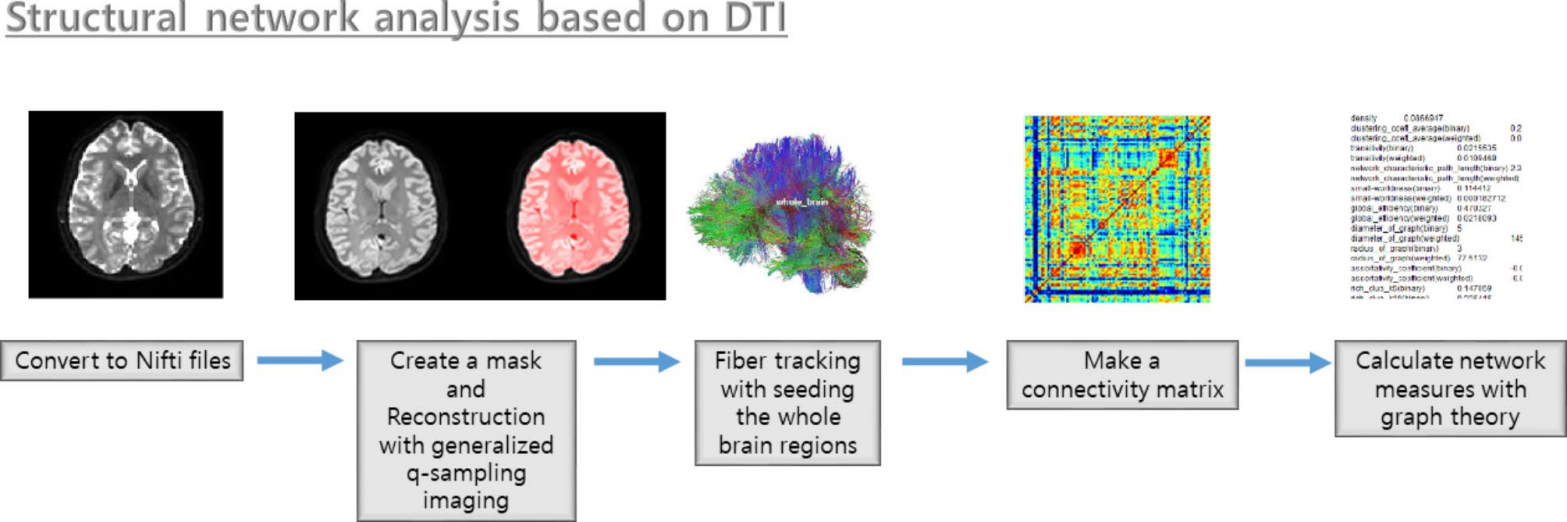
The process for structural network analysis in this study. DTI, diffusion tensor imaging

Using the DSI studio program (https://dsi-studio.labsolver.org), we computed the measures of global and local structural networks in patients with OLE and healthy controls (Fig. 1). The initial steps included opening the source DTI images and creating a mask using thresholding, smoothing, and defragmentation. To address susceptibility artifacts and eddy current distortion, we performed preprocessing with the FSL top-up and eddy. With generalized q-sampling imaging reconstruction to T1-weighted image space, [23] we then generated one fiber orientation per voxel and associated anisotropy and diffusivity measures. This method accounts for individual variations in brain parcellation; however, fiber tracking was performed in the native space. The diffusion data were reconstructed to the subject’s T1-weighted image space so that subsequent analysis could be performed in T1-weighted image coordinates. Fiber tracking was performed by a total of 1,000,000 seeding the entire brain utilizing a deterministic fiber-tracking algorithm [24]. Tracks with length shorter than 60 or longer than 300 mm were discarded. To ensure that the built-in parcellation with the Kleist atlas [25] was registered with the subject data, we performed spatial normalization. The connectivity matrix was calculated by using count of the connecting tract passing through the regions of the Kleist atlas, and 0.001 of the sum was set as the threshold. Graph theory was used to determine the network measures. We selected the weighted measures such that the matrix’s maximum value was one. The global structural network was evaluated using metrics such as the assortative coefficient, mean clustering coefficient, global efficiency, characteristic path length, and small-worldness index, whereas the local structural network was analyzed using the betweenness centrality metric [4, 19, 26, 27]. We further analyzed the local structural network using the automated anatomical labelling 3 atlas (AAL3 atlas) to observe changes in the anatomical topology [28].

### Statistical analysis

Categorical variables including sex were analyzed using the chi-square test and continuous variables including age and network measures were tested using the independent samples t-test. Correlation analysis was conducted using Pearson’s test. All statistical analyses were performed using MedCalc® Statistical Software version 20.014 (MedCalc Software Ltd, Ostend, Belgium; https://www.medcalc.org; 2021). Statistical significance was set at *p* < 0.05. Multiple corrections with Benjamini-Hochberg method to control false discovery rate were applied in the analysis of the global and local structural networks [29]. Categorical variables are presented as frequencies with percentages, and continuous variables are presented as mean values with standard deviation (SD) or median with interquartile range, depending on normal distribution.

## Results

### Clinical characteristics

We enrolled 23 patients with OLE and 42 healthy controls in this study. Table 1 shows the clinical characteristics of the patients with OLE. Age and male sex did not differ between patients with OLE and healthy controls (33.1 vs. 32.2 years, *p* = 0.737; 10/23 (43.5%) vs. 21/42 (50.0%), *p* = 0.737, respectively). Of the 23 patients with OLE, 15 (65.2%) showed visual symptoms as ictal clinical manifestations of epilepsy, and five patients (21.8%) had oculomotor symptoms.

**Table 1:** Clinical characteristics in patients with occipital lobe epilepsy and healthy controls

	Patients with occipital lobe epilepsy (N = 23)	Healthy controls (N = 42)	*p*-value
Mean Age ± SD, years	33.1 ± 15.3	32.2 ± 6.6	0.737
Male/Female, N (%)	10(43.5)/13(56.5)	21(50.0)/21(50.0)	0.617
Median Age of seizure onset, years (interquartile range)	14 (8.3–21.3)		
Initial seizure semiology			
Visual symptoms, N (%)	15 (65.2)		
Oculomotor symptoms, N (%)	5 (21.8)		
Headache, dizziness, nausea, N (%)	3 (13.0)		
Structural lesions on brain MRI, N (%)	0 (0)		
Epileptiform occipital activity on EEG, N (%)	23 (100)		

SD: standard deviation, MRI: magnetic resonance imaging, EEG: electroencephalography

### Global and local structural network

Table 2 presents the results of global structural network analysis. There were significant differences in the global structural network between patients with OLE and healthy controls. The assortativity coefficient (-0.0864 vs. -0.0814, *p* = 0.0214), mean clustering coefficient (0.0061 vs. 0.0064, *p* = 0.0203), global efficiency (0.0315 vs. 0.0353, *p* = 0.0086), and small-worldness index (0.0001 vs. 0.0001, *p* = 0.0175) in patients with OLE was lower than those in the healthy controls. However, the characteristic path length (59.2724 vs. 53.4684, *p* = 0.0120) was higher in patients with OLE than that in healthy controls.

**Table 2:** Differences of the network measures between the groups, which shows altered global structural network in patients with occipital lobe epilepsy

Network measures	Healthy controls (N = 42)		Patients with occipital lobe epilepsy (N = 23)				
	Mean	SD	Mean	SD	Difference	95% CI	*p*-value
Assortativity coefficient	-0.08144	0.00820	-0.08643	0.00811	-0.00499	-0.00920 to -0.00076	*0.0214
Mean clustering coefficient	0.00649	0.00081	0.00617	0.00028	-0.00033	-0.00059 to -0.00005	*0.0203
Global efficiency	0.03532	0.00788	0.03153	0.00339	-0.00380	-0.00659 to -0.00099	*0.0086
Characteristic path length	53.46840	8.45370	59.27240	8.75710	5.80390	1.31880 to 10.28900	*0.0120
Small-worldness index	0.00013	0.00005	0.00011	0.00002	-0.00002	-0.00003 to -0.00000	*0.0175

SD: standard deviation, CI: confidence interval

*With statistical significance

There were also significant differences in the local structural networks between patients with OLE and healthy controls (Suppl. 1). Table 3 presents the regions of the nodes showing significant differences between the groups, even after multiple corrections. The betweenness centrality of the right K1_3a_3b, right K19_3, and right K23_24_26_29_30_31_32_33 decreased, whereas the betweenness centrality of the left K19_1, right K39_1, and right K8_1 increased in patients with OLE compared to healthy controls.

**Table 3:** Network nodes showing significant differences of the betweenness centrality between the patients with occipital lobe epilepsy and healthy controls

Network nodes	Healthy controls (N = 42)		Patients with occipital lobe epilepsy (N = 23)		
**Kleist atlas**					
(function)	Mean	SD	Mean	SD	*p*-value
right K1 3a 3b	42.87	15.93	26.86	13.52	0.0001
(Touch, Pain, Temperature sensation)					
left K19 1	18.49	12.69	33.42	15.63	0.0002
(Calculation, Recognition of numbers, Reading, Visual thinking, Visual recognition)					
right K19 3	56.10	17.85	39.96	15.53	0.0003
(Color and object recognition)					
right K23 24 26 29 30 31 32 33	43.93	17.40	27.87	13.53	0.0001
(Corporeal ego, personal experience and awareness)					
right K39 1	5.50	3.94	10.23	5.25	0.0004
(Constructive action, sensory)					
right K8 1	47.79	11.80	67.89	20.30	0.0001
(Falling and pointing reactions)					
**Automated anatomical labelling 3 atlas**	Mean	SD	Mean	SD	*p*-value
left Posterior cingulate gyrus	29.35	9.64	38.82	18.01	0.0074
left Fusiform gyrus	49.04	14.12	72.97	53.40	0.0078
left Superior temporal gyrus	83.66	20.73	101.79	33.71	0.0092
left Middle temporal gyrus	79.51	20.52	100.43	32.57	0.0023
left Inferior occipital gyrus	20.15	9.61	32.22	23.12	0.0043
left Lingual gyrus	45.34	15.23	66.13	38.66	0.0030
left Superior parietal gyrus	15.10	6.12	23.18	15.51	0.0040
right Posterior cingulate gyrus	22.38	9.23	32.97	21.40	0.0071
right Inferior frontal gyrus, opercular part	45.44	10.59	55.19	18.05	0.0078
right Parahippocampal gyrus	82.11	20.80	98.09	25.83	0.0085
right Postcentral gyrus	29.97	9.17	37.21	11.15	0.0065
right Temporal pole: middle temporal gyrus	65.73	18.99	83.85	34.21	0.0077
right Calcarine fissure and surrounding cortex	41.96	14.34	59.50	37.84	0.0092
right Precentral gyrus	23.74	11.71	41.00	16.10	< 0.0001
right Insula	13.82	4.17	25.59	21.78	0.0011
right Lingual gyrus	31.17	10.85	52.28	37.00	0.0010
right Superior occipital gyrus	32.63	8.89	58.76	55.11	0.0036
right Superior temporal gyrus	57.44	14.83	82.50	55.27	0.0072

SD: standard deviation, CI: confidence interval

**Fig. 2:**
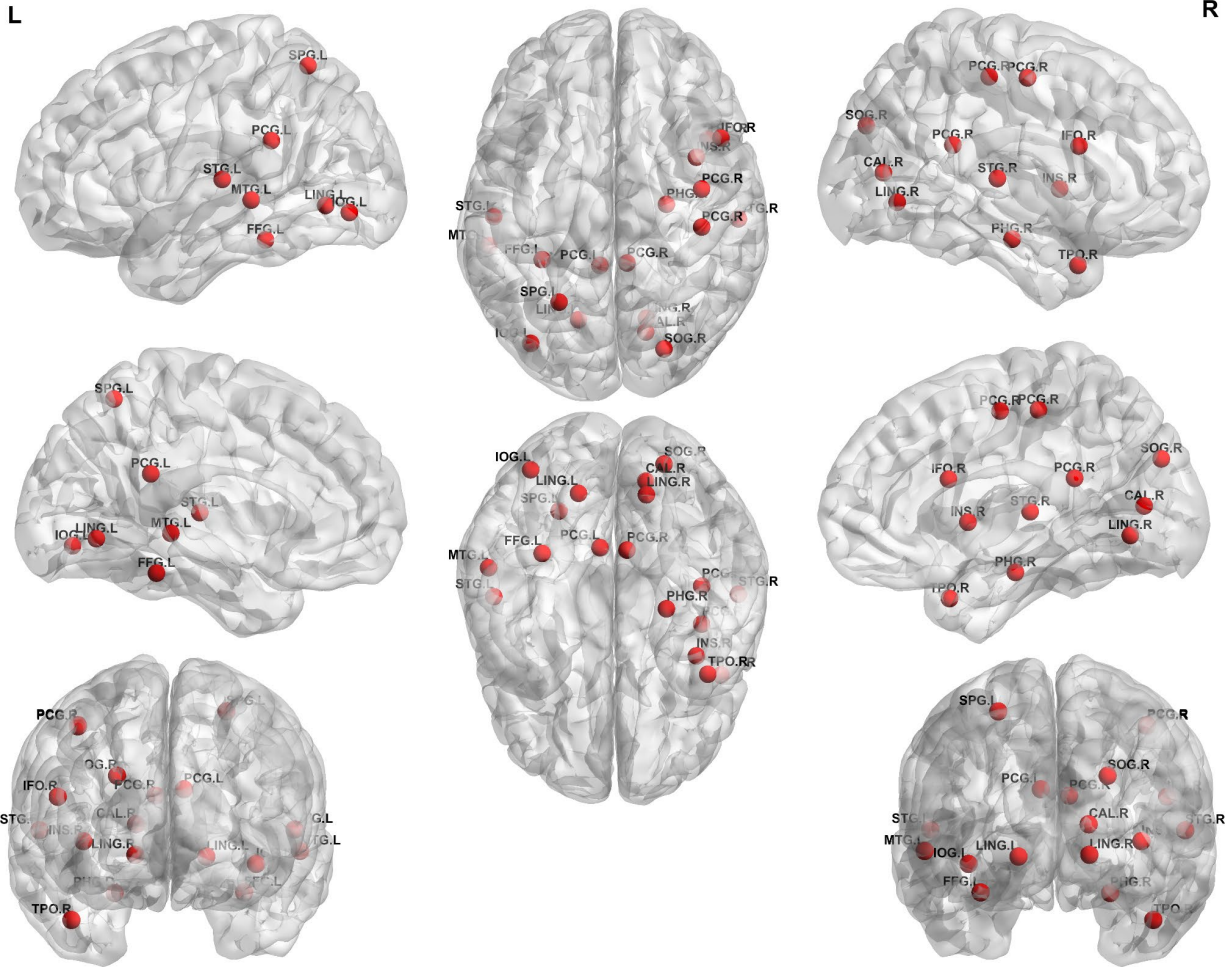
The regions showing significant differences in the local structural networks between the patients with occipital lobe epilepsy and healthy controls The betweenness centrality of the red circles is decreased in patients with occipital lobe epilepsy compared to healthy controls PCG.L: left posterior cingulate, FFG.L: left fusiform gyrus, STG.L: left superior temporal gyrus, MTG.L: left middle temporal gyrus, IOG.L: left inferior occipital gyrus, LING.L: left lingual gyrus, SPG.L: left superior parietal gyrus, PCG.R: right posterior cingulate gyrus, IFO.R: right inferior frontal gyrus opercular part, PHG.R: right parahippocampal gyrus, PCG.R: right postcentral gyrus, TPO.R: right temporal pole middle temporal gyrus, CAL.R: right calcarine fissure and surrounding cortex, PCG.R: right precentral gyrus, INS.R: right insula, LING.R: right lingual gyrus, SOG.R: right superior occipital gyrus, STG.R: right superior temporal gyrus

Figure 2 shows the regions showing significant differences in the local structural networks using AAL3 atlas between the groups. The betweenness centrality of the left posterior cingulate, left fusiform gyrus, left superior temporal gyrus, left middle temporal gyrus, left inferior occipital gyrus, left lingual gyrus, left superior parietal gyrus, right posterior cingulate gyrus, right inferior frontal gyrus opercular part, right parahippocampal gyrus, right postcentral gyrus, right temporal pole middle temporal gyrus, right calcarine fissure and surrounding cortex, right precentral gyrus, right insula, right lingual gyrus, right superior occipital gyrus, and right superior temporal gyrus were decreased in patients with OLE compared to healthy controls (Table 3).

### Correlation analysis between clinical characteristics and structural network

**Fig. 3:**
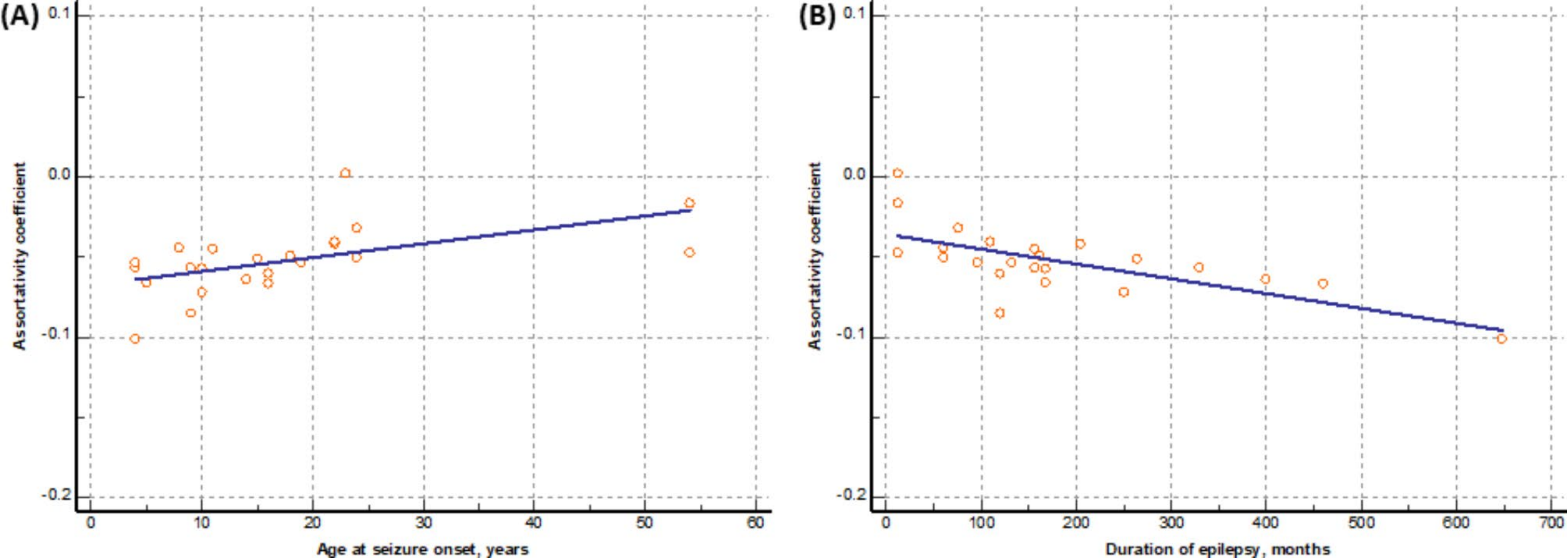
Correlation analysis between clinical characteristics and structural network in patients with occipital lobe epilepsy The assortative coefficient is positively correlated with age at seizure onset (r = 0.531, *p* = 0.019) **(A)**, whereas it is negatively correlated with the duration of epilepsy (r=-0.676, *p* = 0.001) **(B)**

There was a significant correlation between clinical characteristics and the global structural network. The assortative coefficient was positively correlated with age at seizure onset (r = 0.531, *p* = 0.019), whereas it was negatively correlated with the duration of epilepsy (r=-0.676, *p* = 0.001) (Fig. 3). However, the assortative coefficient did not correlate with age (r=-0.144, *p* = 0.510). The other network measures, including the mean clustering coefficient, global efficiency, characteristic path length, and small-worldness index, were not correlated with age (r = 0.080, *p* = 0.716; r = 0.069, *p* = 0.755; r = 0.009, *p* = 0.967; r = 0.122, *p* = 0.580, respectively), age of seizure onset (r = 0.152, *p* = 0.534; r = 0.142, *p* = 0.561; r=-0.082, *p* = 0.738; r = 0.135, *p* = 0.582, respectively), or duration of epilepsy (r=-0.263, *p* = 0.275; r=-0.336, *p* = 0.160; r = 0.347, *p* = 0.145; r=-0.300, *p* = 0.212, respectively).

## Discussion

The main finding of this study was to demonstrate an altered global structural network in patients with OLE compared to that in healthy controls. The assortativity coefficient, mean clustering coefficient, global efficiency, and small-worldness index in patients with OLE was lower than those in the healthy controls, whereas the characteristic path length was higher in patients with OLE than that in healthy controls. We also found that several regions of nodes showed significantly altered local structural networks beyond the occipital lobe in patients with OLE. In addition, the network measure of the assortative coefficient was negatively correlated with the duration of epilepsy.

In a graph theoretical analysis, the mean clustering coefficient is a measure the proportion of connections between its neighbor nodes, which provides insight into the presence of local clustering structures within the graph. The global efficiency is a measure of the efficiency of distant information transfer in a network, and is defined as the inverse of the typical path length between all nodes [19, 26]. The characteristic path length is a measure of the average shortest path length between all pairs of nodes, which quantifies the distance it takes to travel from one node to another node in the graph. The small-worldness index is a measure of the small-network property, and it can be defined as the ratio between the clustering coefficient and average path length [19, 26, 30]. We found that the mean clustering coefficient, global efficiency, and small-worldness index were lower, whereas the characteristic path length was higher in patients with OLE than those in healthy controls. This finding is consistent with the results of previous studies. A previous study in patients with focal epilepsy with normal brain MRI demonstrated that the global efficiency as well as the characteristic path length, mean clustering coefficient, and small-worldness index were decreased compared to those in healthy subjects [31]. Decreased global efficiency and small-worldness index with increased characteristic path length in patients with OLE reflects decreased integration of various brain regions and can be interpreted as a larger pathological epileptic network in the brain, which could be related with the epileptogenesis in OLE. Alternatively, the structural brain network may be damaged by recurrent seizures, resulting in decreased global efficiency [5]. Decreased mean clustering coefficient means that the segregation of network in patients with OLE is reduced, which indicated the nodes tend to form less organization and specialization of the local system [19, 26]. Therefore, from the present findings in this study, it can be confirmed that patients with OLE have a brain network with poor integration and segregation compared to the healthy control, which may be related with pathogenesis in OLE.

Another interesting finding of this study was the negative correlation between the assortative coefficient and the duration of epilepsy in patients with OLE. Assortativity, also known as assortative mixing, is the tendency of network nodes to connect with similar nodes. In a brain network with a high assortative coefficient, nodes tend to connect with other nodes that have similar degree values (assortative network), whereas in a network with a low assortative coefficient, high-degree nodes tend to connect with low-degree nodes (disassortative network) [32]. It is known that numerous technological and biological networks are exemplars of disassortative networks, whereas nearly all social networks are reported to be assortative networks [33]. For example, it is easy to see that a social network has an assortative network. If we look closely at our social life, there are many cases in which people who have a similar degree of connection in society get to know each other. People who are actively social are more likely to know each other. Conversely, looking at photos of fungal strains under a microscope, we can observe that the biological network has a disassortative network. As well as being more susceptible to attack for the network, such as epileptic seizure, disassortative networks appear to be easier to synchronize [34]. Therefore, we found that the longer the duration of epilepsy, the more vulnerable the OLE patient’s brain and the better the excitation brain network. We also previously found that the measure of assortative coefficient based on DTI and graph theory in newly diagnosed epilepsy was associated with the ASM response [8]. The assortative coefficient of ASM good responders was significantly higher than that of ASM poor responders, suggesting that changes in the structural brain network may serve as a biomarker for predicting ASM responses [8]. Whether the assortative coefficient is related to the ASM response in OLE also needs to be studied in the future.

In this study, the Kleist atlas was used for built-in parcellation to analyze alterations in the local structural brain network in patients with OLE. The Kleist atlas describes the anatomical and behavioral characteristics of patients with traumatic and nontraumatic focal brain lesions [25]. Kleist deduced localized cerebral functions based on the loss of function resulting from these lesions. Kleist determined the precise spatial location of the brain lesions post-mortem and correlated them with the patient’s previously documented loss of function [25]. In addition, a recent study showed that most Kleist regions showed large consistency with Brodmann’s atlas [25]. Therefore, we were able to infer abnormal function in patients with OLE, not just the region where the local structural network had an abnormality. Here, we found alterations compared to healthy controls in the local structural network mainly related to the parietal lobe, such as touch and pain sensation and constructive sensory, as well as functions normally performed by the occipital lobe, such as visual recognition and thinking, color, and object recognition. We often observe extra-lesional network abnormalities in patients with epilepsy, and our present study results are not different from those. This phenomenon probably originates in the complex network of the brain. There are many direct and indirect connections between the occipital and parietal lobes. Recent models of visuospatial processing also indicate that the medial parieto-occipital cortex is a key node of the dorsal visual stream [35]. Using a combination of task-evoked activity and resting-state functional MRI, Tosoni et al. demonstrated significant functional specialization and integration within the medial parieto-occipital cortex, a region of the key node in the dorsal visual stream [36]. Therefore, it seems that abnormalities can be observed in nodes that normally function in lobes beyond the occipital lobe in patients with OLE.

In addition to the Kleist atlas, we further analyzed the local structural network using the AAL3 atlas to observe changes in the anatomical topology. We found that the centrality measures were decreased at several nodes located in the frontal, temporal, and parietal lobes beyond occipital lobe in patients with OLE. In focal epilepsy, the appearance of connectivity changes in other lobes beyond the focal epileptogenic lesion was also observed in other studies. A study with patients with temporal lobe epilepsy demonstrated altered hub nodes and changes of the centrality measures in the occipital lobe beyond temporal lobe compared to healthy controls [37]. It was also a consistent finding with previous study demonstrating that focal lesions like stroke had impact on the entire brain and its network properties [38]. In addition, patients with temporal lobe epilepsy with hippocampal sclerosis, which was a typical focal epilepsy, showed low cortical thickness in extratemporal as well as extrahippocampal regions compared to healthy controls, which was demonstrated with the ENIGMA study with large sample size [39]. This findings could suggest that epilepsy is a network disease, and this may also apply to OLE.

This study has several limitations. First, only a small number of patients with OLE were enrolled. However, OLE is a rare disease that accounts for only approximately 5% of focal epilepsy, and we excluded patients with structural lesions because they could affect DTI and significantly affect the structural brain network. Second, because of rapid seizure propagation in OLE from the occipital lobe to the adjacent frontal, temporal, or parietal lobes, as well as the midbrain tegmentum, it is often difficult to delineate the seizure-onset zone in the occipital lobe [21, 40]. Thus, in the absence of occipital lesions detected by imaging studies, it can be difficult to distinguish OLE from other forms of epilepsy. However, we only included patients with OLE who had seizure semiology typical of OLE and ictal EEG showing seizure activity originating in the occipital lobe. Finally, this study had a cross-sectional design, which could not reveal the causal relationship between the altered structural brain network and the pathogenesis of OLE.

## Conclusion

We first evaluated alterations in the structural brain network in patients with OLE. The global efficiency in patients with OLE was lower than that in healthy controls, suggesting decreased integration in the brain network. We also found several regions of nodes that showed significantly altered local structural networks in patients with OLE. This study could suggest that epilepsy is a network disease, and this may also apply to OLE.

## Electronic supplementary material

Below is the link to the electronic supplementary material.

Supplementary Material 1

## Data Availability

The datasets used and/or analysed during the current study are available from the corresponding author on reasonable request.
