# Supplementary material for: Structural brain network analysis in occipital lobe epilepsy
Source: BMC Neurol. 2023 Jul 15;23:268. doi: 10.1186/s12883-023-03326-z (PMC10349483; doi:10.1186/s12883-023-03326-z)
Supplement: Supplementary file 1 — Supplementary Material 1 [file 12883_2023_3326_MOESM1_ESM.docx]

**Supplementary 1.** The differences of the local brain network between the patients with occipital lobe epilepsy and healthy controls

| Network nodes | Patients with occipital lobe epilepsy (N=23) | | Healthy controls (N=42) | |  | | |
| --- | --- | --- | --- | --- | --- | --- | --- |
|  | Mean | SD | Mean | SD | Difference | 95% CI | *p*-value |
| K1_3a_3b_L_Touch_Pain_Temperature_sensation | 14.9175 | 7.6631 | 17.2318 | 7.9245 | 2.3144 | -1.8086 to 6.4373 | 0.2661 |
| K1_3a_3b_R_Touch_Pain_Temperature_sensation | 42.8745 | 15.9272 | 26.8645 | 13.5209 | -16.0100 | -23.4780 to -8.5419 | 0.0001 |
| K10_L_Motor_skill | 46.0089 | 24.8328 | 52.8858 | 23.8402 | 6.8768 | -5.8446 to 19.5982 | 0.2841 |
| K10_R_Motor_skill | 65.0638 | 34.5110 | 75.4231 | 37.5883 | 10.3593 | -8.8825 to 29.6011 | 0.2860 |
| K11_12_L_Personal_and_social_ego | 6.1604 | 12.3151 | 3.7385 | 6.1970 | -2.4219 | -7.0313 to 2.1875 | 0.2976 |
| K11_12_R_Personal_and_social_ego | 1.9892 | 4.0791 | 3.7120 | 7.9370 | 1.7227 | -1.8953 to 5.3407 | 0.3449 |
| K13_L | 15.1024 | 8.6526 | 16.1632 | 7.3197 | 1.0607 | -3.0411 to 5.1625 | 0.6070 |
| K13_R | 12.4859 | 8.5822 | 11.6780 | 8.0318 | -0.8079 | -5.1336 to 3.5178 | 0.7102 |
| K16_L | 20.6313 | 14.1570 | 21.9656 | 15.8860 | 1.3343 | -6.7266 to 9.3952 | 0.7418 |
| K16_R | 17.3126 | 11.6545 | 13.5974 | 6.7381 | -3.7152 | -8.3024 to 0.8721 | 0.1105 |
| K17_1_L_Vision_brightness_colors_forms_movements | 27.0514 | 20.6587 | 31.9698 | 27.6580 | 4.9184 | -8.4986 to 18.3355 | 0.4665 |
| K17_1_R_Vision_brightness_colors_forms_movements | 7.3823 | 8.2222 | 8.2740 | 6.0146 | 0.8916 | -2.7082 to 4.4915 | 0.6223 |
| K17_2_L_Visual_field_lower_quadrant | 15.7518 | 22.3908 | 13.6028 | 13.9424 | -2.1489 | -11.2362 to 6.9383 | 0.6381 |
| K17_2_R_Visual_field_lower_quadrant | 10.1060 | 10.3015 | 10.0907 | 12.0579 | -0.0153 | -6.0617 to 6.0311 | 0.9960 |
| K17_3_L_Visual_field_upper_quadrant | 27.4448 | 20.2868 | 24.0849 | 19.3129 | -3.3599 | -13.6966 to 6.9768 | 0.5182 |
| K17_3_R_Visual_field_upper_quadrant | 14.9016 | 10.7838 | 14.6931 | 19.3424 | -0.2085 | -9.1178 to 8.7009 | 0.9628 |
| K18_1_L_Sense_of_place_Eye_movements_Optic_awareness | 19.9534 | 18.7808 | 23.4360 | 18.4321 | 3.4826 | -6.2769 to 13.2421 | 0.4783 |
| K18_1_R_Sense_of_place_Eye_movements_Optic_awareness | 8.1569 | 6.4973 | 12.8320 | 13.0651 | 4.6751 | -1.2579 to 10.6080 | 0.1203 |
| K18_2_L_Conjugate_downward_eye_movements | 7.6932 | 11.2533 | 8.0151 | 10.6298 | 0.3218 | -5.3836 to 6.0273 | 0.9106 |
| K18_2_R_Conjugate_downward_eye_movements | 5.6054 | 8.4443 | 4.6330 | 7.0916 | -0.9723 | -4.9585 to 3.0138 | 0.6275 |
| K18_3_L_Conjugate_upward_eye_movements | 43.7281 | 23.7393 | 45.8237 | 30.2755 | 2.0956 | -12.7568 to 16.9479 | 0.7789 |
| K18_3_R_Conjugate_upward_eye_movements | 34.4806 | 22.4767 | 34.9127 | 21.9497 | 0.4322 | -11.2100 to 12.0743 | 0.9411 |
| K19_1_L_Calculation_Recognition_of_numbers_Reading_Visual_thinking_Visual_recognition | 18.4933 | 12.6901 | 33.4239 | 15.6253 | 14.9306 | 7.3276 to 22.5336 | 0.0002 |
| K19_1_R_Calculation_Recognition_of_numbers_Reading_Visual_thinking_Visual_recognition | 6.9936 | 7.7325 | 7.7105 | 5.8360 | 0.7169 | -2.7238 to 4.1577 | 0.6785 |
| K19_2_L_Place_memory | 43.7249 | 28.2530 | 45.5021 | 24.7239 | 1.7773 | -11.8869 to 15.4414 | 0.7957 |
| K19_2_R_Place_memory | 30.3039 | 23.9700 | 30.8863 | 20.1929 | 0.5824 | -10.7531 to 11.9178 | 0.9185 |
| K19_3_L_Color_and_object_recognition | 44.4241 | 31.8538 | 34.7357 | 16.6421 | -9.6884 | -21.7636 to 2.3868 | 0.1138 |
| K19_3_R_Color_and_object_recognition | 56.1046 | 17.8544 | 39.9588 | 15.5295 | -16.1458 | -24.6360 to -7.6555 | 0.0003 |
| K2_L_Kinesthetic_sensation | 24.3543 | 11.0056 | 27.4163 | 11.0442 | 3.0621 | -2.7413 to 8.8654 | 0.2956 |
| K2_R_Kinesthetic_sensation | 22.6159 | 22.7322 | 22.0579 | 20.4634 | -0.5579 | -11.7421 to 10.6262 | 0.9209 |
| K20_L_Appreciation_of_sounds_and_music | 27.2992 | 19.8642 | 34.7085 | 22.4290 | 7.4092 | -3.9514 to 18.7699 | 0.1972 |
| K20_R_Appreciation_of_sounds_and_music | 11.9092 | 12.8589 | 15.0066 | 11.0691 | 3.0974 | -3.0611 to 9.2558 | 0.3186 |
| K21_L_Hearing_movements_Acoustic_awareness | 27.1309 | 29.7053 | 23.7856 | 20.5518 | -3.3453 | -15.9955 to 9.3049 | 0.5990 |
| K21_R_Hearing_movements_Acoustic_awareness | 15.7306 | 12.7079 | 16.6005 | 10.6744 | 0.8699 | -5.1295 to 6.8693 | 0.7729 |
| K22a_1_L_Noise_understanding | 10.6044 | 8.8433 | 9.8034 | 10.0495 | -0.8011 | -5.8820 to 4.2799 | 0.7537 |
| K22a_1_R_Noise_understanding | 9.6780 | 9.8740 | 10.0293 | 5.5203 | 0.3514 | -3.4850 to 4.1877 | 0.8553 |
| K22a_2_L_Melody_understanding | 21.2055 | 16.1771 | 18.3406 | 16.9306 | -2.8649 | -11.6397 to 5.9098 | 0.5164 |
| K22a_2_R_Melody_understanding | 14.4940 | 11.3783 | 14.9166 | 9.8974 | 0.4226 | -5.0607 to 5.9058 | 0.8781 |
| K22b_1_L_Word_understanding | 7.6509 | 10.2206 | 6.3675 | 6.8167 | -1.2834 | -5.5609 to 2.9942 | 0.5509 |
| K22b_1_R_Word_understanding | 7.0378 | 8.4462 | 4.1982 | 3.2892 | -2.8395 | -5.7836 to 0.1046 | 0.0584 |
| K22b_2_L_Sentence_understanding | 0.2944 | 0.4086 | 0.2896 | 0.6233 | -0.0048 | -0.2994 to 0.2897 | 0.9739 |
| K22b_2_R_Sentence_understanding | 0.8990 | 1.3250 | 0.4675 | 0.3630 | -0.4314 | -0.8658 to 0.002960 | 0.0515 |
| K23_24_26_29_30_31_32_33_L_Corporeal_ego_personal_experience_awareness | 53.9689 | 51.4072 | 42.9478 | 20.9859 | -11.0211 | -29.1414 to 7.0993 | 0.2287 |
| K23_24_26_29_30_31_32_33_R_Corporeal_ego_personal_experience_awareness | 43.9277 | 17.4038 | 27.8676 | 13.5318 | -16.0601 | -23.8978 to -8.2223 | 0.0001 |
| K25_L | 12.0267 | 14.1250 | 14.2181 | 12.0762 | 2.1913 | -4.5463 to 8.9289 | 0.5180 |
| K25_R | 9.7344 | 14.8402 | 5.7435 | 8.9153 | -3.9910 | -9.9234 to 1.9414 | 0.1836 |
| K27_L | 10.0455 | 11.4872 | 6.1727 | 6.4563 | -3.8728 | -8.3449 to 0.5993 | 0.0884 |
| K27_R | 14.3559 | 15.0414 | 10.8512 | 11.6023 | -3.5047 | -10.2758 to 3.2663 | 0.3048 |
| K28_34_L_Olfactory_recognition | 33.6084 | 18.8289 | 30.2451 | 15.5593 | -3.3633 | -12.1693 to 5.4426 | 0.4481 |
| K28_34_R_Olfactory_recognition | 22.9667 | 19.5734 | 23.5546 | 21.1465 | 0.5879 | -10.2638 to 11.4396 | 0.9141 |
| K35_36_L_Objective_olfactory_movements | 30.6839 | 22.1236 | 26.0394 | 19.8152 | -4.6446 | -15.4957 to 6.2066 | 0.3955 |
| K35_36_R_Objective_olfactory_movements | 11.5446 | 14.5404 | 9.3807 | 10.1528 | -2.1639 | -8.3837 to 4.0559 | 0.4894 |
| K37_L_Name_understanding | 70.5827 | 38.1604 | 61.3404 | 22.5995 | -9.2423 | -24.4078 to 5.9232 | 0.2278 |
| K37_R_Name_understanding | 46.6873 | 27.0697 | 42.7180 | 22.5534 | -3.9693 | -16.6890 to 8.7504 | 0.5350 |
| K38_L | 75.8876 | 40.1077 | 68.2951 | 25.6140 | -7.5925 | -24.0509 to 8.8659 | 0.3600 |
| K38_R | 55.2785 | 29.7495 | 52.8097 | 30.8399 | -2.4688 | -18.5013 to 13.5638 | 0.7593 |
| K39_1_L_Constructive_action_sensory | 9.0849 | 14.9291 | 14.2674 | 12.9840 | 5.1826 | -2.0111 to 12.3763 | 0.1549 |
| K39_1_R_Constructive_action_sensory | 5.4961 | 3.9437 | 10.2284 | 5.2491 | 4.7324 | 2.2267 to 7.2378 | 0.0004 |
| K39_40_L_Body_image_Right-Left_orientation | 5.4487 | 6.1765 | 3.7274 | 3.4181 | -1.7214 | -4.1119 to 0.6692 | 0.1551 |
| K39_40_R_Body_image_Right-Left_orientation | 3.0269 | 3.1165 | 3.3299 | 2.7203 | 0.3031 | -1.2019 to 1.8080 | 0.6887 |
| K4_L_Individual_movements | 58.3642 | 23.6389 | 57.8821 | 18.2504 | -0.4822 | -11.1286 to 10.1643 | 0.9282 |
| K4_R_Individual_movements | 76.4507 | 37.3789 | 77.4482 | 25.0636 | 0.9975 | -14.6854 to 16.6804 | 0.8992 |
| K40_1_L_Individual_consecutive_action | 3.3572 | 3.8845 | 2.5846 | 3.2089 | -0.7726 | -2.5890 to 1.0437 | 0.3984 |
| K40_1_R_Individual_consecutive_action | 2.4086 | 1.8024 | 1.7792 | 1.2436 | -0.6294 | -1.3959 to 0.1372 | 0.1058 |
| K40_2_L_Recognition_by_touch | 3.8096 | 4.2632 | 2.4593 | 1.9031 | -1.3503 | -2.8886 to 0.1881 | 0.0843 |
| K40_2_R_Recognition_by_touch | 1.5312 | 1.3123 | 1.9698 | 1.5473 | 0.4386 | -0.3357 to 1.2130 | 0.2619 |
| K40_3_L_Face_activity_sensory | 2.6266 | 2.6988 | 1.9269 | 1.7393 | -0.6997 | -1.8117 to 0.4122 | 0.2131 |
| K40_3_R_Face_activity_sensory | 0.4095 | 0.4118 | 0.4160 | 0.4878 | 0.0065 | -0.2373 to 0.2503 | 0.9577 |
| K41_42_52_L_Sound_Noise_Loudness_sensation | 2.8696 | 1.8031 | 3.0885 | 2.4025 | 0.2189 | -0.9478 to 1.3857 | 0.7089 |
| K41_42_52_R_Sound_Noise_Loudness_sensation | 2.3825 | 5.1437 | 1.5553 | 1.2291 | -0.8272 | -2.4876 to 0.8332 | 0.3232 |
| K43_L_taste | 2.6678 | 1.9317 | 2.2246 | 1.6673 | -0.4432 | -1.3698 to 0.4834 | 0.3427 |
| K43_R_taste | 1.0007 | 0.7189 | 0.8779 | 0.8045 | -0.1228 | -0.5313 to 0.2857 | 0.5501 |
| K44a_L_word_formation | 10.9517 | 9.9615 | 9.4653 | 6.5544 | -1.4864 | -5.6295 to 2.6567 | 0.4760 |
| K44a_R_word_formation | 11.3051 | 5.5198 | 12.6172 | 7.6669 | 1.3121 | -2.3778 to 5.0019 | 0.4799 |
| K44b_L_Name_speaking_spontaneous | 6.5504 | 6.4082 | 6.8556 | 7.5963 | 0.3052 | -3.4910 to 4.1014 | 0.8728 |
| K44b_R_Name_speaking_spontaneous | 3.9372 | 4.0449 | 4.4771 | 8.1189 | 0.5400 | -3.1476 to 4.2276 | 0.7707 |
| K45_L_Sentence_speaking | 23.8168 | 17.4105 | 26.0387 | 15.9379 | 2.2219 | -6.4330 to 10.8769 | 0.6096 |
| K45_R_Sentence_speaking | 17.8671 | 16.7287 | 27.2442 | 24.7114 | 9.3771 | -2.3703 to 21.1245 | 0.1157 |
| K46_L_Constructive_thinking | 11.3572 | 11.7520 | 13.8097 | 12.4329 | 2.4525 | -3.9692 to 8.8742 | 0.4481 |
| K46_R_Constructive_thinking | 9.6949 | 15.8015 | 17.3355 | 21.1164 | 7.6405 | -2.6073 to 17.8884 | 0.1412 |
| K47_L_sentiment_attitude_mood_affected_actions_perseverance | 27.3797 | 17.5334 | 24.5335 | 11.1546 | -2.8462 | -10.0290 to 4.3366 | 0.4313 |
| K47_R_sentiment_attitude_mood_affected_actions_perseverance | 44.1108 | 27.3591 | 38.3434 | 22.5178 | -5.7675 | -18.5336 to 6.9987 | 0.3700 |
| K5_L | 32.2623 | 14.8012 | 32.3970 | 17.7317 | 0.1348 | -8.7017 to 8.9713 | 0.9758 |
| K5_R | 69.4192 | 43.8987 | 56.7655 | 25.4119 | -12.6537 | -29.9410 to 4.6335 | 0.1485 |
| K6aa_L_Dexterity_Power_perception_Tone_and_sound_formation | 52.7187 | 24.2824 | 50.0773 | 20.9910 | -2.6414 | -14.2999 to 9.0171 | 0.6522 |
| K6aa_R_Dexterity_Power_perception_Tone_and_sound_formation | 83.3913 | 48.4639 | 86.0823 | 27.3352 | 2.6911 | -16.2019 to 21.5840 | 0.7768 |
| K6ab_1_L_Trunk_turning | 27.8951 | 16.1821 | 28.5802 | 21.6428 | 0.6850 | -9.8164 to 11.1865 | 0.8967 |
| K6ab_1_R_Trunk_turning | 48.6823 | 23.4043 | 55.6757 | 24.0659 | 6.9934 | -5.5507 to 19.5374 | 0.2694 |
| K6ab_2_L_Head_turning | 4.9445 | 6.4761 | 4.6982 | 5.1071 | -0.2464 | -3.1969 to 2.7041 | 0.8680 |
| K6ab_2_R_Head_turning | 1.5925 | 2.5620 | 3.0243 | 8.9377 | 1.4318 | -2.4715 to 5.3351 | 0.4662 |
| K7_L_Action_sensory_leg_trunk | 101.0887 | 61.6869 | 112.9951 | 43.2188 | 11.9065 | -14.5245 to 38.3374 | 0.3713 |
| K7_R_Action_sensory_leg_trunk | 106.9073 | 55.3322 | 100.5943 | 34.9852 | -6.3130 | -28.9190 to 16.2930 | 0.5787 |
| K8_1_L_Falling_and_pointing_reactions | 49.5392 | 27.0582 | 52.5290 | 31.5905 | 2.9898 | -12.8623 to 18.8418 | 0.7074 |
| K8_1_R_Falling_and_pointing_reactions | 47.7875 | 11.7977 | 67.8913 | 20.3045 | 17.8019 | 10.8758 to 29.3317 | 0.0001 |
| K8_2_L_Ocular_turning | 4.3042 | 5.0838 | 6.0052 | 5.7153 | 1.7010 | -1.1975 to 4.5996 | 0.2452 |
| K8_2_R_Ocular_turning | 2.3571 | 6.1626 | 3.0427 | 10.2128 | 0.6856 | -4.0736 to 5.4448 | 0.7743 |
| K9_L_Initiative_Awareness_of_effort_and_power | 37.4148 | 31.3366 | 36.7115 | 20.1294 | -0.7033 | -13.5958 to 12.1892 | 0.9135 |
| K9_R_Initiative_Awareness_of_effort_and_power | 52.7858 | 35.2230 | 62.7611 | 41.4990 | 9.9753 | -10.7975 to 30.7480 | 0.3408 |
